# Numerical Investigation of Ferrofluid Preparation during In-Vitro Culture of Cancer Therapy for Magnetic Nanoparticle Hyperthermia

**DOI:** 10.3390/s21165545

**Published:** 2021-08-18

**Authors:** Izaz Raouf, Piotr Gas, Heung Soo Kim

**Affiliations:** 1 Department of Mechanical, Robotics and Energy Engineering, Dongguk University-Seoul, 30 Pildong-ro 1-gil, Jung-gu, Seoul 100-715, Korea; izazraouf@dongguk.edu; 2 Department of Electrical and Power Engineering, AGH University of Science and Technology, Mickiewicza 30 Avenue, 30-059 Krakow, Poland

**Keywords:** cancer therapy, magnetic hyperthermia, magnetic nanoparticles, linear response theory, ferrofluid parameters, induction heating, heat transfer analysis

## Abstract

Recently, *in-vitro* studies of magnetic nanoparticle (MNP) hyperthermia have attracted significant attention because of the severity of this cancer therapy for *in-vivo* culture. Accurate temperature evaluation is one of the key challenges of MNP hyperthermia. Hence, numerical studies play a crucial role in evaluating the thermal behavior of ferrofluids. As a result, the optimum therapeutic conditions can be achieved. The presented research work aims to develop a comprehensive numerical model that directly correlates the MNP hyperthermia parameters to the thermal response of the *in-vitro* model using optimization through linear response theory (LRT). For that purpose, the ferrofluid solution is evaluated based on various parameters, and the temperature distribution of the system is estimated in space and time. Consequently, the optimum conditions for the ferrofluid preparation are estimated based on experimental and mathematical findings. The reliability of the presented model is evaluated via the correlation analysis between magnetic and calorimetric methods for the specific loss power (SLP) and intrinsic loss power (ILP) calculations. Besides, the presented numerical model is verified with our experimental setup. In summary, the proposed model offers a novel approach to investigate the thermal diffusion of a non-adiabatic ferrofluid sample intended for MNP hyperthermia in cancer treatment.

## 1. Introduction

Magnetic nanoparticle (MNP) hyperthermia has a great deal of potential for cancer therapy because of its effectiveness and minimal invasive effects on the healthy tissues surrounding the tumor [1,2,3]. In this therapy, the malignant tissues are damaged with the help of the targeted heat induction caused by nanoparticles in the presence of an alternating current (AC) magnetic field [4,5,6]. Notwithstanding the clinical effectiveness of MNP hyperthermia [7], avoiding the unwanted thermal stress of normal tissues is a significant challenge. The accurate evaluation of the transient and spatial temperature distribution is critical for the clinical applications of MNP hyperthermia [8,9,10]. Hence, *in-silico* studies based on various numerical methods are employed to explore the parameters that optimize the hyperthermia process to evaluate the temperature [7,11,12,13,14] and thermal damage of the tumor containing nanoparticles [15,16]. Many research efforts are currently unde way to synthesize specialized MNPs with various chemical structures and shapes [17,18,19,20] that are suitable for targeted drug delivery [21,22,23], hyperthermia [24,25,26], and photo-thermal procedures [27,28,29]. Magnetic fluids are also being successfully used in some critical industrial applications [30].

*In-vitro* studies of magnetic fluids (MFs) suitable for MNP hyperthermia application have attracted a great deal of attention because of the severity of these therapies in *in-vivo* applications [31,32,33,34]. In view of this, various research works have been conducted to evaluate the ferrofluid behavior to evaluate the AMF-induced heating process of MNP hyperthermia [32,35,36,37,38]. The phenomenon of MF heating under the AC magnetic field is a complex physical effect, where the electromagnetic (EM) energy is converted to power dissipation induced via Brownian and Néel relaxation, and hysteresis losses (in large-sized MNPs) [39,40,41]. The induction heating of MNPs exposed to an applied magnetic field (AMF) is specified as the specific loss power (SLP), also called the specific absorption rate (SAR) [42,43,44], which is a quantity that evaluates the efficiency of nanoparticles in transforming EM energy into power. The SLP can be calculated by calorimetric and magnetic methods [7,45,46]. It has been recorded that the SLP values are influenced by multiple parameters of the calorimetric setup, such as the volume of the MF sample, the shape of the Eppendorf tube, the thermo-physical properties of the ferrofluid system, the position of the temperature sensor, and the external conditions [26,47,48]. In addition to the SLP parameter, the intrinsic loss power (ILP) is an additional parameter used to evaluate the magnetic fluid hyperthermia (MFH) and ferrofluid heating efficiency under various experimental conditions of amplitude and frequency of the applied field [49]. Significantly, the ILP parameter depends inversely on the quadratic of the magnetic field strength and the frequency of AMF [50]. As this, theoretically, does not depend on the product *f* × *H*^2^ (A^2^/m^2^/s), it seems to be better for comparing various exposure conditions of tested MF samples. The transformation of EM energy is a complex function of the magnetic field strength, electromagnetic field (EMF) frequency, and the concentration of MNPs [51]. In this regard, Lanier et al. [52] listed different types of MNPs parameters and investigated the correlation between the properties of MNPs and the SLP and ILP values. Castellanos-Rubio et al. [53] presented the effect of MNPs in distilled water, agar, and cell culture media to evaluate the effectiveness of hyperthermia. The magnetite MNPs showed higher values of SLP in water. In [43], the authors proposed simplified models for the determination of the SLP (SAR) of magnetic fluid samples based on the specific heat or volumetric heat capacity of water. Osacia and Cacciola [54] investigated the influence of a nanoparticle coating on SLP-based heat generation during MNP hyperthermia. Importantly, the coupled electro-thermal models were studied to demonstrate the importance of heat losses due to the water-cooled coils for MFH purposes [55,56,57]. However, despite the recent advance in the development of ferrofluid evaluation for MNP/AMF hyperthermia, the heating efficiency of MF samples has been investigated under adiabatic conditions in insulated containers made of Styrofoam [58,59,60]. In view of this, we have recently proposed a comprehensive model that evaluates the potential effect of heat loss from a ferrofluid sample placed in a polystyrene tube of a given thickness [14]. However, the proposed model is only applicable to the *in-vitro* investigations of the ferrofluid system and cannot be used to prepare the MFs for further experiments and *in-vivo* application because the applied frequency exceeds the safety limit of hyperthermia applications [7]. To the best of the authors’ knowledge, no comprehensive work has been dedicated to ferrofluid preparation in a non-adiabatic environment in an *in-vitro* culture for cancer therapy, which is known as magnetic nanoparticle (MNP) hyperthermia. Hence, there is a need to find a model of this kind that can be used to evaluate all parameters that affect the thermal behavior of ferrofluid samples for the *in-vivo* applications of MNP hyperthermia.

This study presents a numerical approach based on experimental and analytical analyses to investigate the parameters involved in affecting the MF samples designed for MNP hyperthermia applications. In view of this, the MNP hyperthermia parameters are directly correlated with the temporal and spatial temperature distribution. The thermal behavior of the ferrofluid sample is evaluated for different frequencies, strengths of the AC magnetic field, and MNP concentrations. In addition, the effect of temperature loss is investigated for the thickness of the tube wall and the convection heat loss from the ferrofluid system to the surrounding. The presented numerical model is verified with our experimental setup [14]. A comparative analysis is carried out between the magnetic and calorimetric methods for both the SLP and ILP calculations. Finally, the presented model is shown to be a reliable tool that is applicable for the ferrofluid preparation of MNP hyperthermia applications in cancer therapy.

## 2. Methodology

**Figure 1:**
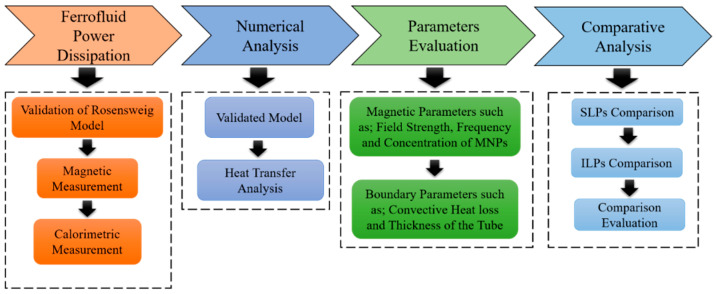
Schematic of the proposed methodology.

Figure 1 shows a flow diagram of the approach presented in this study. Initially, the magnetite (Fe_3_O_4_) MNPs in the sample are dispersed in distilled water and then subjected to the AC magnetic field. The magnetic power losses in the form of SLP and ILP parameters are measured using linear response theory (LRT) and utilized for numerical modeling by evaluating various parameters that include those obtained from the LRT basis and boundary-based parameters. A thermal analysis is carried out for different strengths and frequencies of the applied magnetic field and MNP concentrations, as employed for the LRT parameters. To consider the effect of the power loss from the boundaries, the impacts of the tube wall thickness and convection heat losses are investigated. The calorimetric method is used, and the SLP/ILP values are determined from a numerical analysis of the temperature curves based on the initial slope method (ISM). Finally, a comparative analysis is carried out for the LRT-based magnetic method and the calorimetric method.

## 3. Mathematical Modeling

### 3.1. Experimental Model

In the present study, our previous experimental setup is considered as a benchmark [14]. The Fe_3_O_4_ MNPs were commercially obtained from Micromod GmBH (catalog no. nanomag-D-Spio, #79-00-102), which has a stock concentration of 25 mg/mL. The ferrofluids used in this study were prepared by diluting the stock solution of MNPs (25 mg/mL) with water into various concentrations, and the solutions were used for experiments. The magnetic field was measured by locating a magnetic field probe in the center of the coil chamber in the absence of a magnetic fluid sample. For the measurement of temperature in the magnetic fluid, the magnetic fluid sample was inserted into the center region of the coil chamber while the fiber optic temperature sensor was positioned inside the eight-well stripe. Initially, we intended to place the fiberoptic temperature sensor in the center region of the well, but there was a technical difficulty in precisely locating and fixing the sensor; hence, the temperature sensor was positioned to offset the central position.

### 3.2. Magnetic Model

The power dissipation of the ferrofluid sample was modeled under the AMF using the LRT formulation developed by Rosensweig [46]. This well-known model was implemented and further extended to our problem. The volumetric power *p* (W/m^3^) dissipated in a unit volume of MNPs, as well as SLP (W/kg) and ILP (nH∙m^2^/kg) parameters, respectively, were calculated using the following equations [42,45,46,61]:

(1) $$p=\mu_{0}\pi \chi^{\prime \prime }fH^{2}$$

(2) $${\mathrm{SLP}}_{1}=\frac{p}{\rho_{\mathrm{MNPs}}}=\frac{p}{c_{\mathrm{MNP}}}\phi_{v\mathrm{MNP}\;}$$

(3) $${\mathrm{ILP}}_{1}=\frac{{\mathrm{SLP}}_{1}}{fH^{2}}$$

where μ_0_ = 4π∙10^−7^ H/m represents the permeability of free space, *H* (A/m) is the AMF strength, *f* (Hz) is the frequency of the magnetic field, *χ*’’ is the out-of-phase component of the complex magnetic susceptibility *χ* = *χ*’ − j*χ*’’ for the MF, *c*_MNPs_ (mg/mL) is the concentration of nanoparticles, and *ϕ_v_*_MNPs_ stands for the volume fraction of MNPs in the sample. The imaginary part of the complex magnetic susceptibility can be expressed as [46,62]:

(4) $$\chi^{\prime \prime }=\frac{2\pi f\tau }{1+{\left(2\pi f\tau \right)}^{2}}\chi_{0}$$

where *χ*_0_ represents the static equilibrium magnetic susceptibility, and *τ* (s) is the effective relaxation time. The *χ*_0_-term can be calculated as [7,46]:

(5) $$\chi_{0}=\chi_{i}\frac{3}{\xi }\left(\cot h\xi -\frac{1}{\xi }\right)$$

where *ξ* means the dimensionless Langevin parameter of magnetic–thermal quantity, and $\chi_{i}\;$ is the initial susceptibility; these parameters can be determined as [7,46]:

(6) $$\xi =\frac{\mu_{0}M_{s}V_{\mathrm{MNP}}H}{3k_{B}T}$$

(7) $$\chi_{i}=\frac{\mu_{0}\phi_{v\mathrm{MNPs}}M_{d}^{2}V_{m}}{3k_{B}T}$$

where *M*_s_ and *M*_d_ (A/m) are the saturation and domain magnetization of the MNPs, respectively, k_B_ = 1.38 × 10^−23^ J/K represents the Boltzmann constant, and *T* (K) is the absolute temperature. The effective relaxation time *τ* (s) of MNPs can be determined in terms of two fundamental mechanisms for the orientation of a magnetic particle in external AMF; namely, the Brownian and Néel relaxations. The Néel relaxation *τ*_N_ (s) takes place due to the reorientation of the magnetization vector inside the magnetic core against an energy barrier. On the other hand, the Brownian relaxation *τ*_B_ (s) is caused by particle reorientation as a whole. The Néel relaxation time exponentially increases with the magnetic volume *V*_m_ (m^3^) of MNPs; however, the Brownian relaxation time is linearly dependent on the hydrodynamic volume of magnetic particles [46]. The expressions for the Néel and Brownian relaxation times, as well as the effective relation time, are given by [7,46,49]:

(8) $$\tau_{N}=\frac{\sqrt{\pi }}{2}\tau_{0}\frac{\exp\left(\Gamma \right)}{\Gamma^{1/2}}$$

(9) $$\tau_{B}=\frac{3\eta V_{h}}{k_{B}T}$$

(10) $$\tau ={\left(\frac{1}{\tau_{B}}+\frac{1}{\tau_{N}}\right)}^{-1}$$

where *τ*_0_ = 10^−9^ s is the time constant called the attempt time, *η* (Pa∙s) is the viscosity of the ferrofluid, and *V*_h_ (m^3^) is the hydrodynamic volume of the MNP. The parameter (*Γ*) can be defined as [34,51]:

(11) $$\Gamma =\frac{KV_{m}}{k_{B}T}$$

where *K* denotes the anisotropy constant of a magnetic nanoparticle, and *V*_m_ (m^3^) is the volume of its magnetic core, given by the formula:

(12) $$V_{m}=\frac{4\pi }{3}R^{3}$$

where *R* is the radius of the magnetic particle. The hydrodynamic volume is a function of the non-magnetic hydrodynamic layer thickness *δ* (m) of MNPs and can be determined from [7,46]:

(13) $$V_{h}=\frac{4\pi }{3}{\left(R+\delta \right)}^{3}={\left(1+\delta /R\right)}^{3}V_{m}$$

### 3.3. Calorimetric Model

The calorimetric heating measurement methods were able to determine the values of the SLP and ILP parameters. Multiple techniques could be used to find the power dissipation using the temperature curves of ferrofluid samples [7]. The initial slope method is commonly used to empirically determine the heat losses in magnetic fluid (MF). The following expression can be used to find the SLP and ILP values [43,45,48]:

(14) $${\mathrm{SLP}}_{2}=C_{\mathrm{MF}}\frac{\Delta T}{\Delta t}\frac{m_{\mathrm{MF}}}{m_{\mathrm{MNPs}}}$$

(15) $${\mathrm{ILP}}_{2}=\frac{{\mathrm{SLP}}_{2}}{fH^{2}}$$

where *m*_MF_ and *C*_MF_ (J/kg/K) represent the mass and specific heat capacity of the magnetic fluid, respectively. Moreover, *m*_MNPs_ (kg) is the mass of MNPs, and Δ*T/*Δ*t* (K/s) corresponds to the temperature increment calculated for the initial 20 s of AMF exposure [7,48]. Table 1 specifies the parameters used in measuring power dissipation.

### 3.4. Effective Parameters

The thermal behavior of ferrofluid samples is affected by various parameters, such as the thermo-physical properties of MNPs, water, and the polystyrene tube containing the ferrofluid sample [63]. The ferrofluid sample is a mixture of MNPs and water. Hence, the thermo-physical properties of the aqueous suspension of MNPs depend on the individual properties of the constituents of the MF sample. The expressions for the effective ferrofluid properties, such as their density *ρ*_MF_ (kg/m^3^), specific heat capacity *C*_MF_ (J/kg/K), thermal conductivity *k*_MF_ (W/m/K), and viscosity *η*_MF_ (Pa∙s), respectively, are given by [43,45,48,64,65]:

(16) $$\rho_{\mathrm{MF}}=\phi_{v\mathrm{MNPs}}\rho_{\mathrm{MNPs}}+\phi_{vH_{2}O}\rho_{H_{2}O}$$

(17) $$C_{\mathrm{MF}}=\phi_{m\mathrm{MNPs}}C_{\mathrm{MNPs}}+\phi_{mH_{2}O}C_{H_{2}O}$$

(18) $$k_{\mathrm{MF}}=\left[\frac{k_{\mathrm{MNPs}}+2k_{H_{2}O}+2\left(k_{\mathrm{MNPs}}-k_{H_{2}O}\right)\phi_{v\mathrm{MNPs}}}{k_{\mathrm{MNPs}}+2k_{H_{2}O}-2\left(k_{\mathrm{MNPs}}-k_{H_{2}O}\right)\phi_{v\mathrm{MNPs}}}\right]k_{H_{2}O}$$

(19) $$\eta_{\mathrm{MF}}=\eta_{H_{2}O}/{\left(1-\phi_{v\mathrm{MNPs}}\right)}^{2.5}$$

where *ϕ_v_*_H2O_ and *ϕ_m_*_H2O_ are the volume and mass fractions of water, respectively, and *ϕ_v_*_H2O_ represents the mass fraction of MNPs immersed in aqueous solution. Table 2 summarizes the individual and combined thermo-physical properties of the magnetite MNPs, water, ferrofluid sample, and polystyrene tube, respectively.

### 3.5. Numerical Modeling

Finite element analysis (FEA) was carried out via the ABAQUS simulation package. The optimized Case IV was adopted from our previous studies and describes the effect of heat losses from the ferrofluid system to the surroundings [14]. The following assumptions were considered in the presented model:

- The nanoparticles were homogenously distributed in the MF sample;
- A continuous heat flux was considered from the system to the surroundings;
- The initial temperature of the MF sample was assumed to be 21 °C;
- The convection heat coefficient value was assumed to be 10 W/m^2^/K;
- The volumetric power generation *p* (W/m^3^) was considered as an input parameter to the numerical model and determined using Equation (1).

In view of the optimized Case IV, an interaction was assumed between the outer side of the ferrofluid (master surface) and the inner side of the tube wall (slave surface). An effect of the tube thickness on the heat loss from the ferrofluid sample, and ultimately the thermal behavior of the sample, was assumed. In addition, the effects of convective and radiative heat transfer on the boundaries of the ferrofluid system were investigated. The thermal distribution could be determined from the diffusion heat equation [49]:

(20) $$\rho_{\mathrm{MF}}C_{\mathrm{MF}}\frac{\partial T}{\partial t}=\nabla \cdot \left(k_{\mathrm{MF}}\nabla T\right)+p$$

The initial and boundary conditions are given as follows [14]:

(21) $$T\left(r,\phi ,z,0\right)=T_{0}$$

(22) $$n\cdot \left(-k_{\mathrm{MF}}A\nabla T\left(r,\phi ,z,t\right)\right)=hA\left(T\left(r,\phi ,z,t\right)-T_{\infty }\right)+\sigma \varepsilon A\left(T^{4}\left(r,\phi ,z,t\right)-T_{\infty }^{4}\right)$$

where **n** means the normal vector, *T*_0_ (K) is the initial temperature, *T*∞ (K) represents the surrounding air temperature, *A* (m^2^) is the surface area of the ferrofluid system, *σ* = 5.67 × 10^−8^ W/m^2^/K^4^ is the Stefan–Boltzmann constant, and *ε* is the emissivity. Table 3 describes the scheme of the ferrofluid system dimensions (see Supplementary Information).

## 4. Results and Discussions

### 4.1. Parameters Obtained from the LRT-Bsed Magnetic Method

#### 4.1.1. Applied Magnetic Field Strength

**Figure 2:**
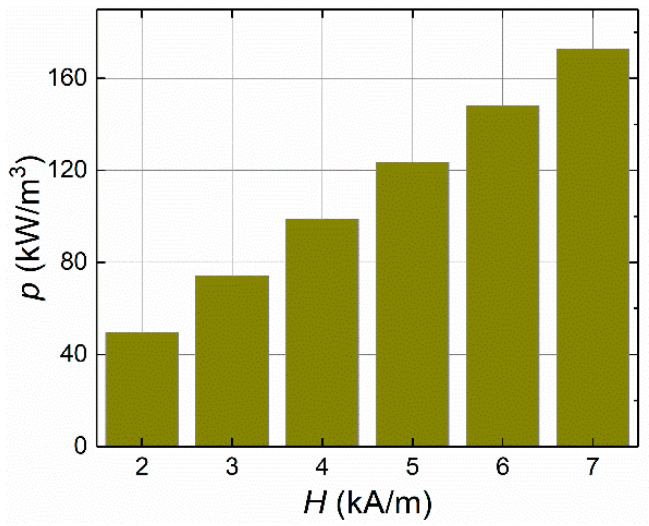
The influence of the AMF strength on the dissipated power (*p*) at an MNP concentration of *c*_MNPs_ = 4 mg/mL and applied frequency *f* = 50 kHz.

The AC magnetic field strength plays a crucial role in the heating phenomenon of the ferrofluid sample. The LRT-based magnetic method was used to estimate the optimum value of AMF strength that could dissipate the maximum power for hyperthermia applications. In view of this, the AMF distribution was found to be directly related to the temperature distribution, both in space and time, for the ferrofluid sample. The volumetric power dissipation was measured for a series of AMF intensities under the limit value for hyperthermia applications (where the product of *H* and *f* was assumed to be between (4.85 and 8.5) × 10^8^ A/m/s and, individually, *H* was assumed to be up to 15 kA/m) [7]. For example, the influence of the AMF strength for a series of values *H*_1_, *H*_2_, *H*_3_, *H*_4_, *H*_5_, and *H*_6_ = 2, 3, 4, 5, 6, and 7 kA/m, respectively, was investigated at a fixed MNP concentration of *c*_MNPs_ = 4 mg/mL and applied frequency of *f* = 50 kHz. Figure 2 shows that the volumetric heat generation revealed a linear relationship with the AMF strength. Interestingly, for *H*_1_, *H*_2_, *H*_3_, *H*_4_, *H*_5_, and *H*_6_ = 2, 3, 4, 5, 6, and 7 kA/m, respectively, the MNPs dispersed in the ferrofluid samples generated power dissipation values at 49.2, 73.9, 98.6, 123.4, 148.1, and 172.8 kW/m^3^, respectively.

**Figure 3:**
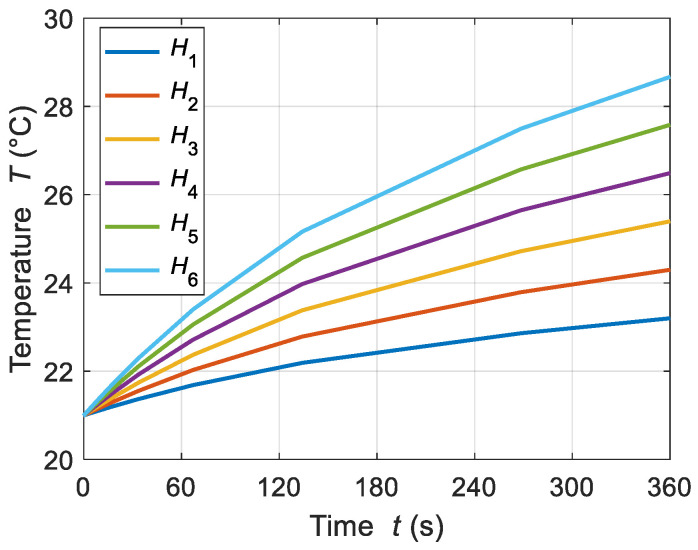
Transient temperature distributions of the ferrofluid sample for different AMF strengths of *H*_1_, *H*_2_, *H*_3_, *H*_4_, *H*_5_, and *H*_6_ = 2, 3, 4, 5, 6, and 7 kA/m, respectively, at an MNP concentration *c*_MNPs_ = 4 mg/mL and frequency of applied field *f* = 50 kHz.

**Figure 4:**
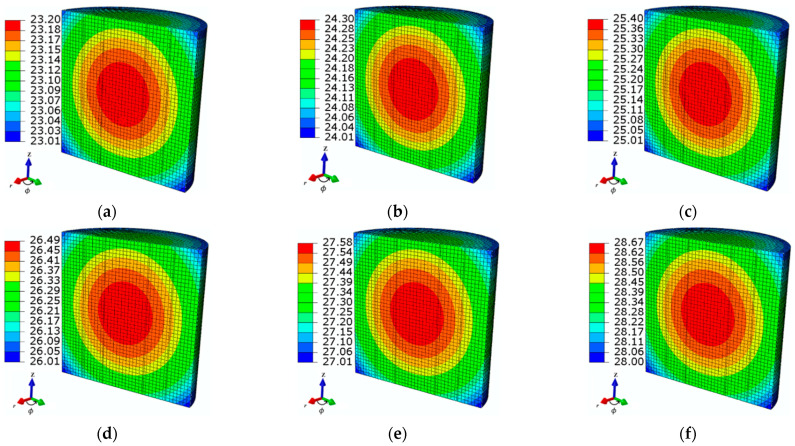
The steady-state spatial temperature distributions of tested ferrofluid samples observed at time *t* = 360 s for different AMF strength values: (**a**) *H*_1_ = 2 kA/m, (**b**) *H*_2_ = 3 kA/m, (**c**) *H*_3_ = 4 kA/m, (**d**) *H*_4_ = 5 kA/m, (**e**) *H*_5_ = 6 kA/m, and (**f**) *H*_6_ = 7 kA/m, at an MNP concentration *c*_MNPs_ = 4 mg/mL and applied frequency *f* = 50 kHz.

The thermal behavior of ferrofluid samples in both time and spatial frames was analyzed by the FEM-based model. In this regard, the ferrofluid system temperature was directly correlated to the AMF strength. The presented model shows an effective increment in the temperature for the tested MF sample. Figure 3 shows the linear relationship observed between the time-dependent temperature increments and the AMF strength values. The temperature increased from 21 to 23.1, 21 to 24.3, and 21 to 25.4 °C, for AMF strengths at *H*_1_, *H*_2_, and *H*_3_ = 2, 3, and 4 kA/m, respectively. A similar trend was observed for the higher values of AMF: *H*_4_, *H*_5_, and *H*_6_ = 5, 6, and 7 kA/m, respectively. Despite the transient temperature distribution, the steady-state spatial temperature distribution was estimated from the ferrofluid sample for different values of AMF strength (see Figure 4).

#### 4.1.2. Frequency of the Applied Magnetic Field

**Figure 5:**
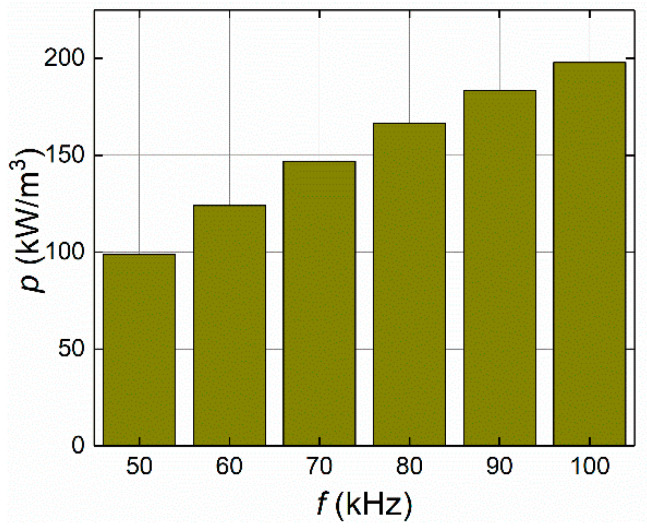
The influence of the frequency values on volumetric power (*p*) levels at the given MNP concentration *c*_MNPs_ = 4 mg/mL and applied AMF strength value *H* = 4 kA/m.

The frequency of the applied magnetic field is one of the key parameters that influences the AMF heating of ferrofluid samples designed for hyperthermia applications. A suitable frequency range was estimated using the LRT-based magnetic method, which generated proper values for the power dissipation of MNPs. The effects of a series of frequency values were considered for the ferrofluid sample heating: *f*_1_, *f*_2_, *f*_3_, *f*_4_, *f*_5_, and *f*_6_ = 50, 60, 70, 80, 90, and 100 kHz, respectively. Figure 5 shows the correspondence of the applied frequencies with the volumetric power dissipation levels for the fixed values of AMF strength of *H* = 4 kA/m and an MNP concentration of *c*_MNPs_ = 4 mg/mL. Note that the frequency of the applied magnetic field shows a linear relationship with the power dissipation levels.

**Figure 6:**
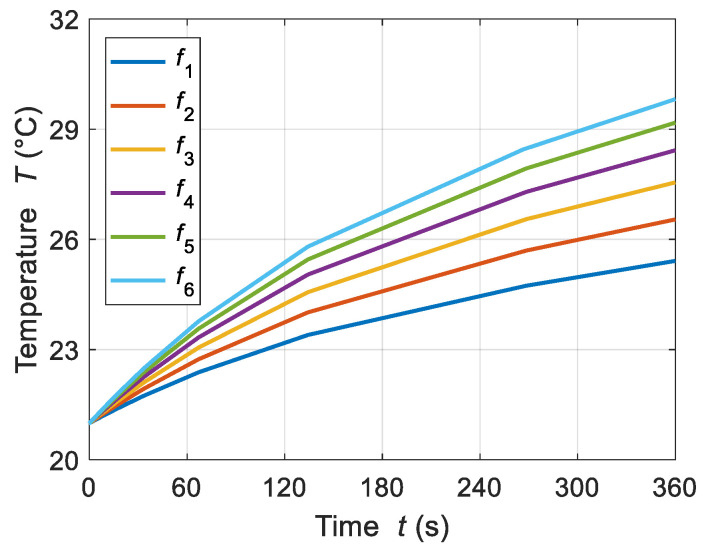
Transient temperature distributions of the ferrofluid samples for various frequencies of applied magnetic field *f*_1_, *f*_2_, *f*_3_, *f*_4_, *f*_5_, and *f*_6_ = 50, 60, 70, 80, 90, and 100 kHz, respectively, at a given MNP concentration *c*_MNPs_ = 4 mg/mL and applied AMF value *H* = 4 kA/m.

**Figure 7:**
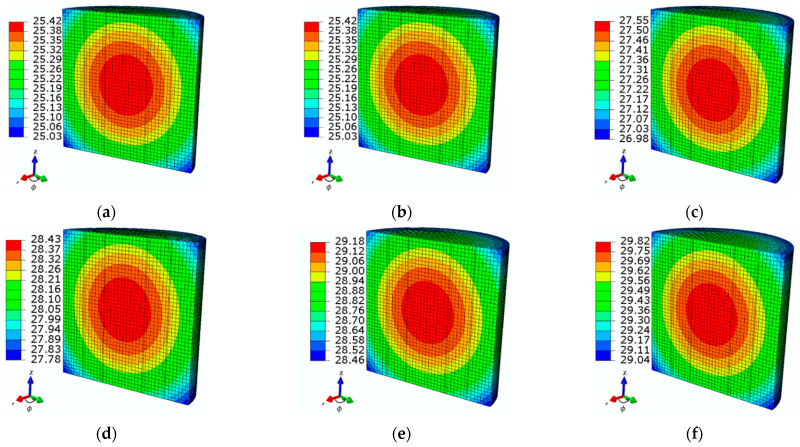
The steady-state spatial temperature distributions of the tested ferrofluid samples observed at time *t* = 360 s for various values of applied frequency: (**a**) *f*_1_ = 50 kHz, (**b**) *f*_2_ = 60 kHz, (**c**) *f*_3_ = 70 kHz, (**d**) *f*_4_ = 80 kHz, (**e**) *f*_5_ = 90 kHz, and (**f**) *f*_6_ = 100 kHz at the given MNP concentration *c*_MNPs_ = 4 mg/mL and applied AMF value *H* = 4 kA/m.

Figure 6 and Figure 7 show the transient and spatial temperature distributions of the ferrofluid samples for different frequencies, respectively. Note that for the series of frequency values equal to *f*_1_, *f*_2_, *f*_3_, *f*_4_, *f*_5_, and *f*_6_ = 50, 60, 70, 80, 90, and 100 kHz, respectively, the recorded changes in temperature increments ∆*T* were 4.4, 5.5, 6.5, 7.4, 8.1, and 8.8 °C, respectively, starting from the baseline MF sample temperature of 21 °C.

#### 4.1.3. MNP Concentrations

**Figure 8:**
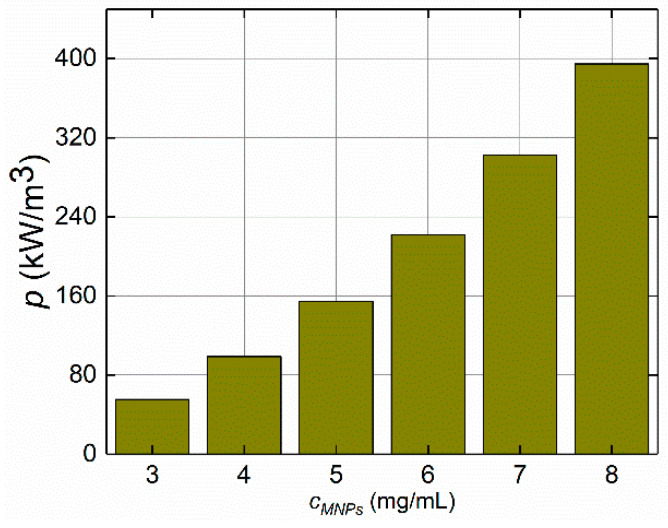
The influence of the MNP concentration levels on volumetric power dissipation (*p*) at the given value of AMF strength *H* = 4 kA/m and applied frequency *f* = 50 kHz.

In addition to the AMF strength and frequency, the MNP concentration is a crucial parameter that influences the MNP heating phenomenon. During AMF/MNP hyperthermia, the proper selection of MNP concentration is key to avoiding the excessive heating of MF samples. For that purpose, various concentrations of MNP were studied to evaluate the effect of MNP accumulation on the power dissipation levels. The impact of six different MNP concentration values of *c*_MNPs1_, *c*_MNPs2_, *c*_MNPs3_, *c*_MNPs4_, *c*_MNPs5_, and *c*_MNPs6_ = 3, 4, 5, 6, 7, and 8 mg/mL, respectively, at a fixed applied frequency of *f* = 50 kHz and given AMF strength value of *H* = 4 kA/m was studied for the tested ferrofluid samples. Note that the thermo-physical properties of the MF sample were affected by the MNP concentration, as studied in Section 3.3 (Table 2). In addition, Figure 8 shows the association of the MNP concentration with the dissipated power by the MNPs in the ferrofluid sample. The MNP concentration showed a linear trend with the dissipated power elevation. In the case of MNP concentrations of 3, 4, and 5 mg/mL, the dissipated power values were 55.5, 98.7, and 154.2 kW/m^3^, respectively.

**Figure 9:**
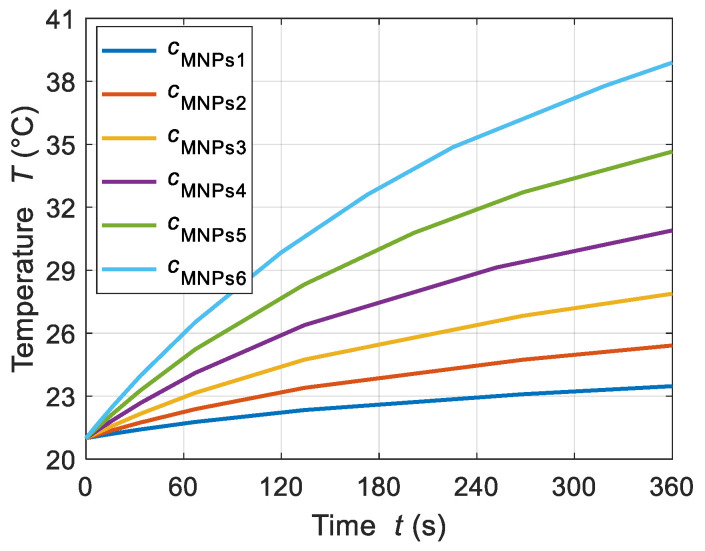
Temperature increments for ferrofluid samples for various MNP concentration levels *c*_MNPs1_, *c*_MNPs2_, *c*_MNPs3_, *c*_MNPs4_, *c*_MNPs5_, and *c*_MNPs6_ = 3, 4, 5, 6, 7, and 8 mg/mL, respectively, at the given value of AMF strength *H* = 4 kA/m and applied frequency *f* = 50 kHz.

The transient and steady-state spatial temperature distributions were estimated using the calculated power dissipation levels in the MNPs. The temperature behavior of the ferrofluid samples showed a linear trend with the growing concentrations of MNPs. In regard to this, higher power was dissipated as the number of the MNPs increased per unit volume of the MF sample; ultimately, the temperature of the sample increased. Figure 9 shows the temperature increments from the initial temperature of 21–23.5, 25.4, 27.8, 30.1, 34.7, and 38.9 °C for concentrations of MNPs of 3, 4, 5, 6, 7, and 8 mg/mL, respectively.

**Figure 10:**
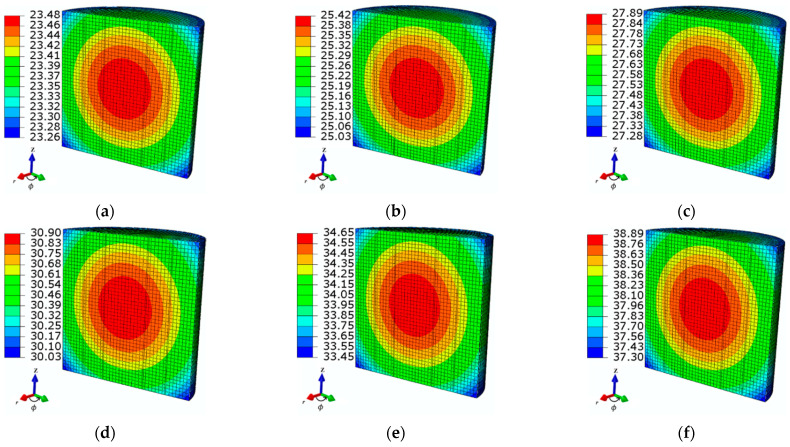
The steady-state spatial temperature distributions of the ferrofluid samples observed at time *t* = 360 s for various MNP concentration levels: (**a**) *c*_MNPs1_ = 3 mg/mL, (**b**) *c*_MNPs2_ = 4 mg/mL, (**c**) *c*_MNPs3_ = 5 mg/mL, (**d**) *c*_MNPs4_ = 6 mg/mL, (**e**) *c*_MNPs5_ = 7 mg/mL, and (**f**) *c*_MNPs6_ = 8 mg/mL at the given AMF value *H* = 4 kA/m and applied frequency *f* = 50 kHz.

In addition, Figure 10 shows the spatial temperature distribution of the ferrofluid sample for various MNP concentrations. The temperature was observed to decrease slightly towards the boundaries because of the heat loss to the surroundings.

### 4.2. Boundary Parameters

#### 4.2.1. Tube Thickness

**Figure 11:**
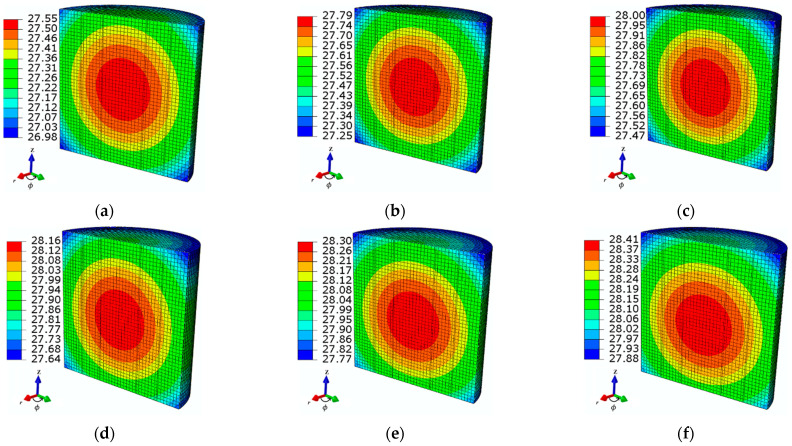
The steady-state spatial temperature distributions of the ferrofluid samples observed at time *t* = 360 s for different tube wall thicknesses: (**a**) *d*_1_ = 0.55 mm, (**b**) *d*_2_ = 1 mm, (**c**) *d*_3_ = 1.5 mm, (**d**) *d*_4_ = 2 mm, (**e**) *d*_5_ = 2.5 mm, and (**f**) *d*_6_ = 3 mm, at a given MNP concentration *c*_MNPs_ = 4 mg/mL, AMF value *H* = 4 kA/m, and applied frequency *f* = 70 kHz.

The tube wall thickness also influenced the *in-vitro* setup of the ferrofluid sample. As a result, the temperature increment of the test MNPs was affected by changing the thickness of the Eppendorf tube wall. In order to evaluate the effect of tube thickness, various tube thicknesses were considered in the numerical model: *d*_1_, *d*_2_, *d*_3_, *d*_4_, *d*_5_, and *d*_6_ = 0.55, 1, 1.5, 2, 2.5, and 3 mm. It is observed that increasing the tube thickness also elevated the ferrofluid temperature, as shown in Figure 11. In support of this, the thickness of the tube wall behaved as a thermal resistance that decreased the heat transfer rate; consequently, the sample temperature increased. In the analyzed cases, the observed temperature increments were 27.5, 27.8, 28, 28.2, 28.3, and 28.4 °C for wall tube thicknesses *d*_1_–*d*_6_, respectively.

**Figure 12:**
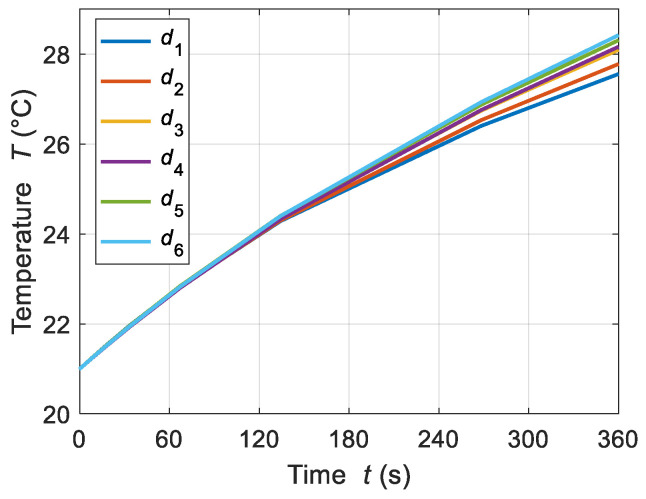
Temperature increments for ferrofluid samples for different values of wall tube thicknesses: *d*_1_ = 0.55 mm, *d*_2_ = 1 mm, *d*_3_ = 1.5 mm, *d*_4_ = 2 mm, *d*_5_ = 2.5 mm, and *d*_6_ = 3 mm, at a given MNP concentration *c*_MNPs_ = 4 mg/mL, AMF strength *H* = 4 kA/m, and applied frequency *f* = 70 kHz.

In view of the transient behavior of temperature, it was observed that the *d*-dependent temperature variation was under 1 °C for all of the analyzed cases. Hence, the transient temperature distributions were not considered for various thicknesses of the tube wall. Figure 12 shows the transient temperature distribution for different wall thickness.

#### 4.2.2. Convective Heat Loss

**Figure 13:**
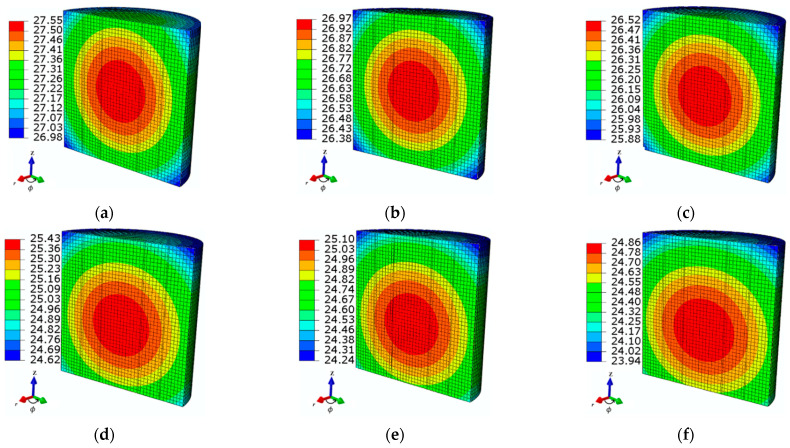
The steady-state spatial temperature distributions of the ferrofluid samples observed at time *t* = 360 s for various convective heat coefficient elevations: (**a**) *h*_1_ = 10 W/m^2^/K, (**b**) *h*_2_ = 20 W/m^2^/K, (**c**) *h*_3_ = 30 W/m^2^/K, (**d**) *h*_4_ = 40 W/m^2^/K, (**e**) *h*_5_ = 50 W/m^2^/K, and (**f**) *h*_6_ = 60 W/m^2^/K, at the given MNP concentration *c*_MNPs_ = 4 mg/mL, AMF strength *H* = 4 kA/m, and applied frequency *f* = 70 kHz.

**Figure 14:**
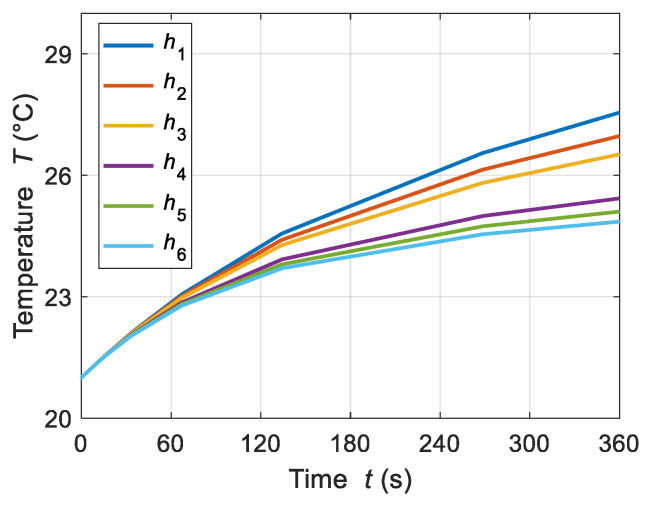
The transient temperature distributions of the ferrofluid samples for various convective heat coefficients elevations: *h*_1_ = 10 W/m^2^/K, *h*_2_ = 20 W/m^2^/K, *h*_3_ = 30 W/m^2^/K, *h*_4_ = 40 W/m^2^/K, *h*_5_ = 50 W/m^2^/K, and *h*_6_ = 60 W/m^2^/K, at the given MNP concentration *c*_MNPs_ = 4 mg/mL, AMF strength *H* = 4 kA/m, and applied frequency *f* = 70 kHz.

The behavior of the convective heat transfer also affects the temperature distribution of the hyperthermia system. To study the effect of convective heat transfer, various convection coefficient levels were considered from natural convection to forced convection, with values from *h*_1_ to *h*_6_ = 10 to 20 W/m^2^/K, in increments of 10 W/m^2^/K. Figure 13 shows the spatial temperature distributions of the presented cases. By increasing the *h*-parameter, a slight change in the temperature increment was evident. In addition, increasing the convective heat coefficient values led the temperature pattern to tend toward the bottom surface because of the heat transfer from the top surface of the ferrofluid system. Figure 14 shows the transient temperature distributions for a series of different convective heat coefficient values.

This demonstrates that the temperature distributions were almost the same for the initial 60 s of MFH treatment. Later on, the transient temperature curves showed a differentiating behavior, and as the convection heat loss increased, they decreased. The measured temperatures were 27.5, 26.9, 26.5, 25.4, 25.1, and 24.8 °C for convection heat coefficients of 10, 20, 30, 40, 50, and 60 W/m^2^/K, respectively. 

### 4.3. Comparitive Analysis

**Figure 15:**
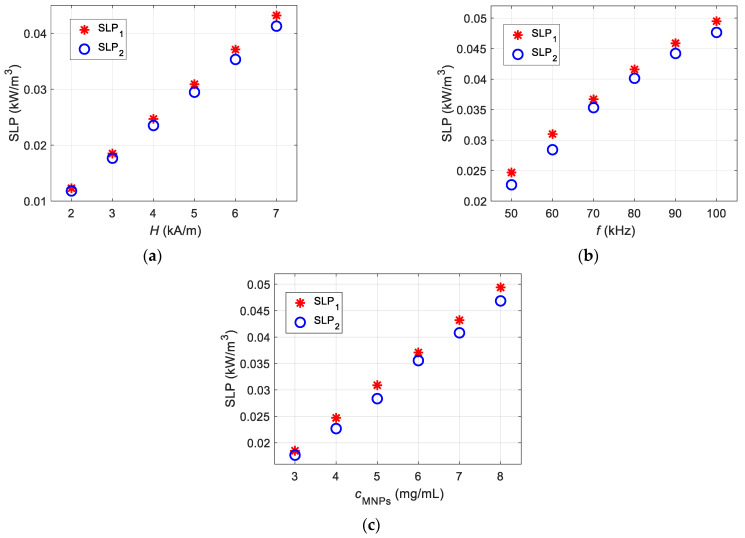
The comparative analysis of the SLP calculation based on the magnetic (SLP_1_) and calorimetric (SLP_2_) methods for multiple presented cases: (**a**) different AMF strength values, (**b**) different frequencies, and (**c**) different MNP concentration levels.

Figure 15 shows the comparison of the magnetic and calorimetric methods to increase the reliability of the presented model. Figure 15a compares both procedures for the six selected AMF strength levels. Note that both techniques show a good correlation. The maximum value of relative error of 5% is observed for an AMF *H* = 6 kA/m. A maximum relative lag at 9% is observed for the calorimetric method over the magnetic method. Figure 15b compares the series of selected frequency values. Similarly, the calorimetric process slightly lags (maximum relative error of 9% for *f* = 60 kHz) behind the LRT-based magnetic method. The slight deviation might be due to the initial slope calculation. However, the recorded variation is at an acceptable level (error is less than 10%) [25]. In addition, a similar trend is observed for the various MNP concentrations presented in Figure 15c.

**Figure 16:**
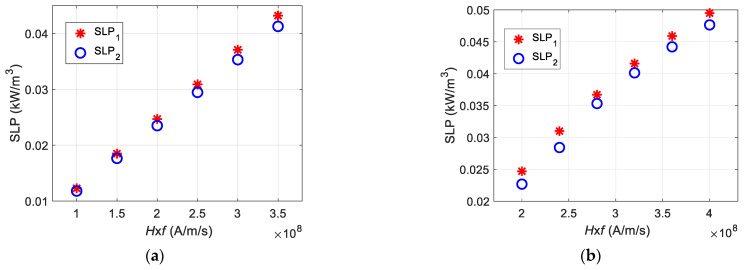
The comparative analysis of SLPs with the product of magnetic field strength and frequency (*H* × *f*) observed for (**a**) *H*_1_, *H*_2_, *H*_3_, *H*_4_, *H*_5_, and *H*_6_ = 2, 3, 4, 5, 6, and 7 kA/m, respectively, at *f* = 50 kHz; (**b**) *f*_1_, *f*_2_, *f*_3_, *f*_4_, *f*_5_, and *f*_6_ = 50, 60, 70, 80, 90, and 100 kHz, respectively, at *H* = 4 kA/m.

Figure 16 shows the relationship of the SLP values obtained from the LRT with respect to the combined effect of the field strength and frequency (*H* × *f*). Figure 16a shows the SLP values, which reveal a linear trend with varying field strengths: *H*_1_, *H*_2_, *H*_3_, *H*_4_, *H*_5_, and *H*_6_ = 2, 3, 4, 5, 6, and 7 kA/m, respectively, at *f* = 50 kHz. Similarly, Figure 16b shows the direct relationship of SLP values with the *H* × *f* product, such as *f*_1_, *f*_2_, *f*_3_, *f*_4_, *f*_5_, and *f*_6_ = 50, 60, 70, 80, 90, and 100 kHz, respectively, at *H* = 4 kA/m.

The relative percentage of errors (RE) between the SLP and ILP values are measured using the following expressions:

(23) $$\mathrm{RE}=\frac{{\mathrm{SLP}}_{1}-{\mathrm{SLP}}_{2}}{{\mathrm{SLP}}_{1}}\times 100\;\mathrm{or}\;\mathrm{RE}=\frac{{\mathrm{ILP}}_{1}-{\mathrm{ILP}}_{2}}{{\mathrm{ILP}}_{1}}\times 100$$

The above expressions give the same output because of the linear relationship between the SLP and ILP parameters. The maximum RE is observed to be equal to 8.29% for the specific case of the parameters *f* = 60 kHz, *c*_MNPs_ = 4 mg/mL, and *H* = 4 kA/m, and this is considered as an acceptable range (the error is less than 10%) [25]. For more details see Supplementary Information. In recent research work, the correlation analysis of the SLPs measured and calculated the results of two distinct methods [25]; however, the presented model is an adiabatic model, and thus the potential effect of heat loss across the boundaries of the ferrofluid is not evaluated. The present study proposes a novel approach to consider the impact of heat loss across the boundaries of the ferrofluid. The overall thermal response of the ferrofluid can be evaluated based on the input parameters. 

## 5. Conclusions

In the current study, a comprehensive model was presented for ferrofluid preparation in MNP hyperthermia applications for cancer therapy. The ferrofluid sample was evaluated under non-adiabatic conditions, and the effects of heat losses were evaluated. In order to evaluate the *in-vitro* model for cancer therapy, the optimized parameters of magnetic field strengths and applied frequency (below the threshold product of *H × f* = 8 × 10^8^ A/m/s) were adopted. The parameters from the LRT-based magnetic method, such as the magnetic field strength, frequency, and MNP concentrations, were investigated and optimized. It is observed that the selected AMF and frequency produced a sufficient heating effect for ferrofluid samples heated in non-adiabatic conditions. In addition, the MNP concentration showed a differentiating impact on the temperature increment of the MF sample. Moreover, the boundary parameters, such as the wall tube thickness and convective heat transfer, also slightly affected the temperature distribution of the ferrofluid system. Hence, these parameters should be considered for the *in-vitro* applications of magnetic hyperthermia. To evaluate the reliability of the presented MFH model, the LRT-based magnetic method was implemented [61]. The presented numerical model was verified with our experimental setup-based calorimetric model [14]. In addition, the reliability of our model could be justified by correlating two different methods of magnetic and calorimetric methods for the SLP and ILP calculations. In summary, the presented model is a trustworthy tool that provides novel information and can be used to prepare ferrofluid samples for MNP hyperthermia. The presented study can be extended for *in-vivo* culture in tumor or human organ modeling.

## Figures and Tables

**Table 1:** Parameters used in the measurement of power dissipation [5,7,52].

Parameters	Description	Values
*R*	Radius of magnetic particle	15 nm
*δ*	Non-magnetic thickness of MNP	2 nm
*M*_d_	Domain magnetization	4.46 × 10^8^ A/m
*T*	Ambient temperature	310 K
k_B_	Boltzmann constant	1.38 × 10^−23^ J/k
μ_0_	Permeability of free space	4π × 10^−7^ H/m
*K*	Magnetic anisotropy constant	4.41 × 10^4^
*H*	Magnetic field strength	(2, 3, 4, 5, 6, & 7) kA/m
*f*	Magnetic field frequency	(50, 60, 70, 80, 90, & 100) kHz
*c*_MNPs_	Concentration of MNPs	(3, 4, 5, 6, 7, & 8) mg/mL
τ_0_	Attempt time constant	10^−9^ s

**Table 2:** Properties of all the materials employed in the MFH calculations [14,66,67,68,69].

Material	*c*_MNPs_ (mg/mL)	*ρ* (kg/m^3^)	*C* (J/kg/K)	*k* (W/m/K)	*η* (Pa∙s)	*ε* (–)
Magnetite	–	5180	670	40	–	–
Water	–	1000	4178	0.6	8.90 × 10^−4^	0.97
Magnetic Fluid (MF)	2	1001.6	4171	0.601	8.91 × 10^−4^	–
	4	1003.2	4164	0.602	8.92 × 10^−4^	–
	6	1004.8	4157	0.602	8.93 × 10^−4^	–
	8	1006.5	4150	0.603	8.94 × 10^−3^	–
	10	1008.1	4143	0.604	8.95 × 10^−3^	–
Plastic tube (polystyrene)	–	55	1210	0.030	–	0.82

**Table 3:** Demonstration of the ferrofluid system dimensions in the numerical modeling.

Parameters	Dimensions
Volume of the magnetic fluid, *V*_MF_	200 μL
Height of the tube, *h*	6.42 mm
Inner diameter of the tube, *d*_in_	6.30 mm
Tube thickness, *d*	0.55 mm
Tube external diameter, *d*_ex_	7.40 mm

## Data Availability

Not applicable.

## Supplementary Materials

The following are available online at https://www.mdpi.com/article/10.3390/s21165545/s1, Figure S1: Representation of (**a**) the ferrofluid system, and (**b**) schematic of the system dimensions: height of the tube (*h*), and its internal diameter (*d*_in_), Figure S2: Comparison of the ILP calculations based on the magnetic (ILP_1_) and calorimetric (ILP_2_) methods for the multiple presented cases: (**a**) different AMF strength values, (**b**) different frequencies, and (**c**) different MNP concentration levels, Figure S3: Comparison of the ILP with the product of the magnetic field strength and frequency (*H × f*) observed for: (**a**) *H*_1_, *H*_2_, *H*_3_, *H*_4_, *H*_5_, and *H*_6_ = 2, 3, 4, 5, 6, and 7 kA/m, respectively, at f = 50 kHz; (**b**) *f*_1_, *f*_2_, *f*_3_, *f*_4_, *f*_5_, and *f*_6_ = 50, 60, 70, 80, 90, and 100 kHz, respectively, at *H* = 4 kA/m, Table S1: Comparative analysis of the SLP and ILP values between the magnetic measurement and calorimetric methods.

## Abbreviations

**Abbreviation**	**Explanation**
AMF	Applied magnetic field
AC	Alternating current
EM/EMF	Electromagnetic/electromagnetic field
FEA	Finite element analysis
FEM	Finite element method
ILP	Intrinsic loss power
LRT	Linear response theory
MF/MFs	Magnetic fluid/magnetic fluids
MFH	Magnetic fluid hyperthemria
MNP/MNPs	Magnetic nanoparticle/magnetic nanoparticles
RE	Relative error
SLP	Specific loss power
