# Supplementary material for: Numerical Investigation of Ferrofluid Preparation during In-Vitro Culture of Cancer Therapy for Magnetic Nanoparticle Hyperthermia
Source: Sensors (Basel). 2021 Aug 18;21(16):5545. doi: 10.3390/s21165545 (PMC8402254; doi:10.3390/s21165545)
Supplement: Supplementary file 1 [file sensors-21-05545-s001.zip › sensors-1335806-supplementary.pdf]

sensors

MDPI

Supplementary Information

Numerical Investigation of Ferrofluid Preparation during In-Vitro Culture of Cancer Therapy for Magnetic Nanoparticle Hyperthermia

Izaz Raouf [1], Piotr Gas [2,*] and Heung Soo Kim [1,*]

[1] Department of Mechanical, Robotics and Energy Engineering, Dongguk University-Seoul, 30 Pildong-ro 1-gil, Jung-gu, Seoul 100-715, Korea; izazraouf@dongguk.edu
[2] Department of Electrical and Power Engineering, AGH University of Science and Technology, Mickiewicza 30 Avenue, 30-059 Krakow, Poland
* Correspondence: piotr.gas@agh.edu.pl (P.G.), heungsoo@dgu.edu (H.S.K.)

1. Numerical modeling

The considered model including dimensions is presented in Figure S1.

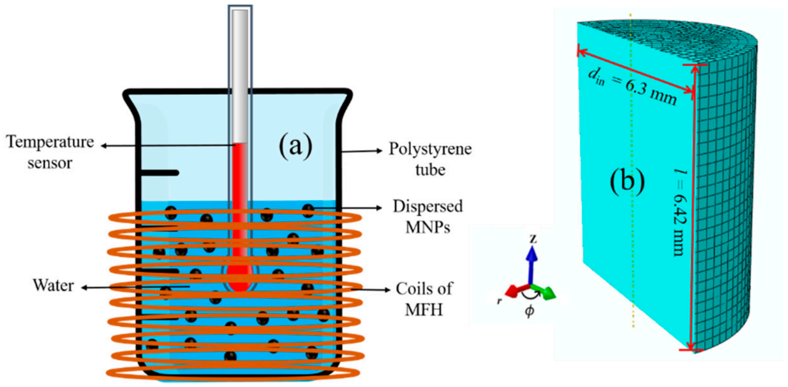

(**a**) (**b**)

Figure S1. Representation of (**a**) the ferrofluid system, and (**b**) schematic of the system dimensions: height of the tube (h), and its internal diameter (d_{in}).

2. ILP Analysis

To evaluate the reliability of the presented study, the ILP values of magnetic measurements were compared with the calorimetric measurements (see Figure S2). Increasing the AMF strengths dramatically decreased the ILP values, and as remarked, the two methods show a reliable correspondence, as shown in Figure S2(a). Figure S2(b) compares the ILP values for the series of magnetic field frequencies. As expected, the ILP values are strongly independent of the selected frequencies. A substantial agreement is observed between the ILP values of both methods. Similarly, comparison of the ILP values for the series of various MNPs concentrations, shown in Figure S2(c) proves the reliability of the presented model for all tested ferrofluid samples. Finally, Table S1 compares the precise SLP and ILP values.

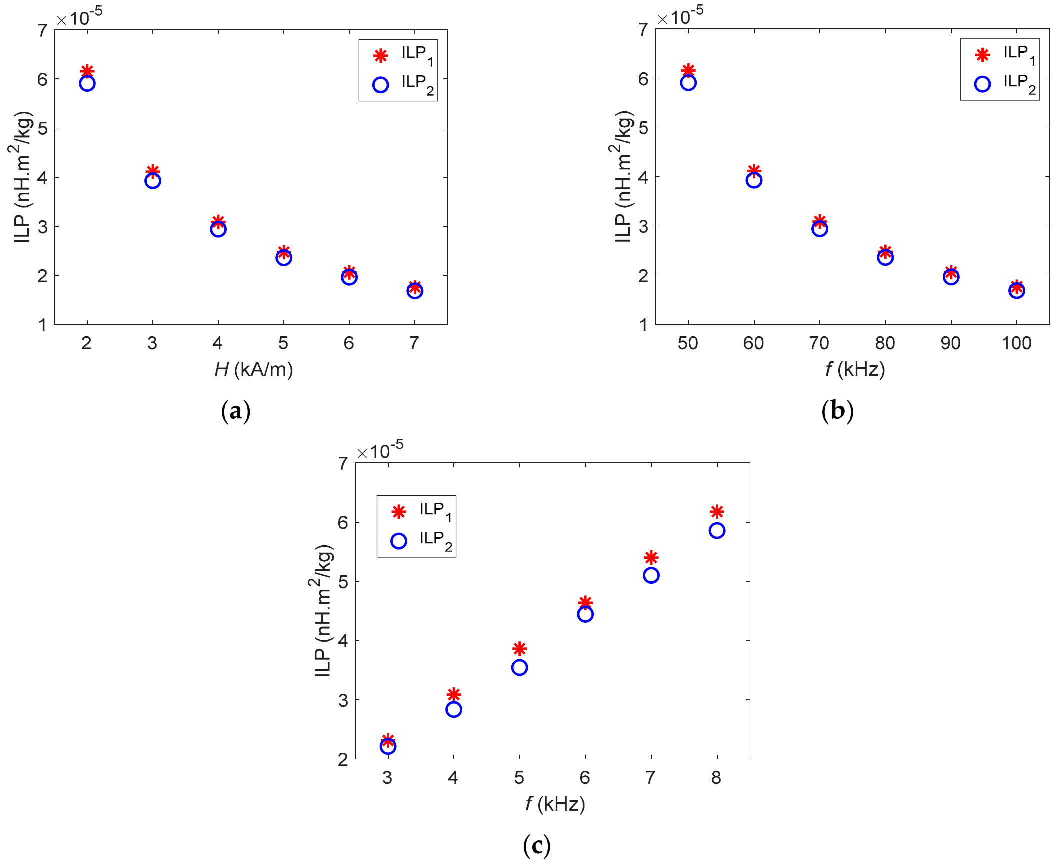

Figure S2. Comparison of the ILP calculations based on the magnetic (ILP_1) and calorimetric (ILP_2) methods for the multiple presented cases: (**a**) different AMF strength values, (**b**) different frequencies, and (**c**) different MNP concentration levels.

Figure S3 shows the relationship of the ILP parameters obtained from the LRT with the *H*×*f* product. This shows that as the AMF strength changes, the ILP value effectively varies (Figure S3(a)). However, Figure S3(b) shows only a slight ILP variation by changing the frequency values.

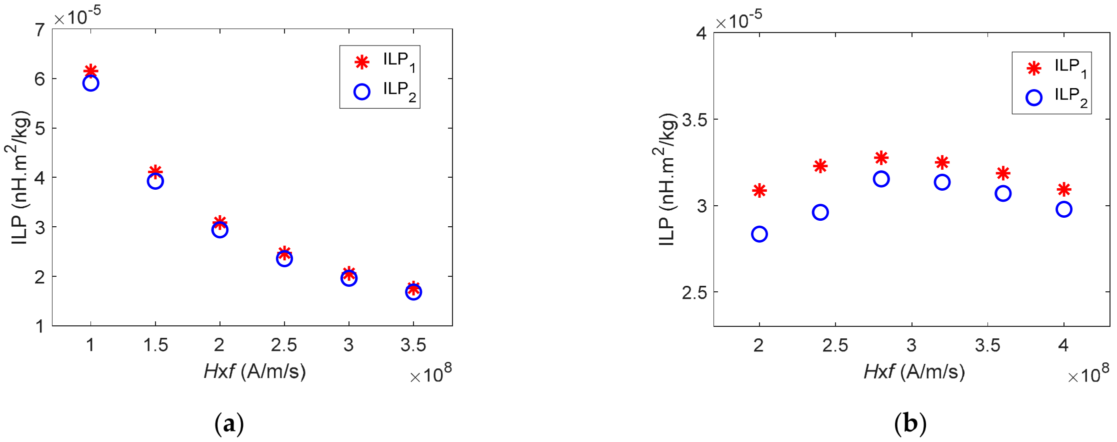

Figure S3. Comparison of the ILP with the product of the magnetic field strength and frequency (*H*×*f*) observed for: (**a**) (H_1, H_2, H_3, H_4, H_5, and H_6) = (2, 3, 4, 5, 6, and 7) kA/m, respectively, at f = 50 kHz); (**b**) (f_1, f_2, f_3, f_4, f_5, and f_6) = (50, 60, 70, 80, 90, and 100) kHz, respectively, at H = 4 kA/m.

3. Comparative Analysis

Table S1. Comparative analysis of the SLP and ILP values between the magnetic measurement and calorimetric methods.

**Parameters and their values**		**Magnetic method SLP_1** (W/g)	**Calorimetric method SLP_2** (W/g)	**Magnetic method $ILP_1 \times 10^{-5}$** (nH·m²/kg)	**Calorimetric method $ILP_2 \times 10^{-5}$** (nH·m²/kg)	**Relative % error RE** [%]
Magnetic field strength H (kA/m)	2	12.3	11.8	6.15	5.91	3.98
	3	18.5	17.6	4.11	3.93	4.52
	4	24.7	23.5	3.09	2.94	4.79
	5	30.9	29.4	2.47	2.36	4.61
	6	37.1	35.3	2.06	1.96	4.78
	7	43.2	41.2	1.76	1.69	4.35
Magnetic field frequency f (kHz)	50	24.7	22.6	3.09	2.84	8.17
	60	31.0	24.8	3.23	2.96	8.29
	70	36.7	35.3	3.28	3.15	3.74
	80	41.6	40.1	3.25	3.14	3.52
	90	45.9	44.2	3.19	3.07	3.68
	100	49.5	47.6	3.09	2.98	3.71
MNPs concentration c_{MNPs} (mg/mL)	3	18.5	17.6	2.31	2.21	4.34
	4	24.7	22.6	3.08	2.83	8.17
	5	30.9	28.3	3.86	3.54	8.25
	6	37.1	35.5	4.63	4.44	4.18
	7	43.2	40.8	5.40	5.10	5.53
	8	49.4	46.8	6.18	5.85	5.15
